# Urinary Microbial and Metabolomic Profiles in Kidney Stone Disease

**DOI:** 10.3389/fcimb.2022.953392

**Published:** 2022-09-05

**Authors:** Hong Gao, Jiaqiong Lin, Fu Xiong, Zuhu Yu, Shilei Pan, Yuxin Huang

**Affiliations:** ^1^ Shenzhen Hospital, University of Chinese Academy of Sciences, Shenzhen, China; ^2^ Affiliated Dongguan Maternal and Child Healthcare Hospital, Southern Medical University, Dongguan, China; ^3^ Department of Medical Genetics/Experimental Education/Administration Center, School of Basic Medical Sciences, Southern Medical University, Guangzhou, China; ^4^ Department of Fetal Medicine and Prenatal Diagnosis, Zhujiang Hospital, Southern Medical University, Guangzhou, China; ^5^ Zhujiang Hospital, Southern Medical University, Guangzhou, China

**Keywords:** nephrolithiasis, metabolome, microbiome, biomarker, urine

## Abstract

**Background:**

Kidney stones or nephrolithiasis is a chronic metabolic disease characterized by renal colic and hematuria. Currently, a pathogenetic mechanism resulting in kidney stone formation remains elusive. We performed a multi-omic study investigating urinary microbial compositions and metabolic alterations during nephrolithiasis.

**Method:**

Urine samples from healthy and individuals with nephrolithiasis were collected for 16S rRNA gene sequencing and liquid chromatography-mass spectroscopy. Microbiome and metabolome profiles were analyzed individually and combined to construct interactome networks by bioinformatic analysis.

**Results:**

Distinct urinary microbiome profiles were determined in nephrolithiasis patients compared with controls. Thirty-nine differentially abundant taxa between controls and nephrolithiasis patients were identified, and Streptococcus showed the most significant enrichment in nephrolithiasis patients. We also observed significantly different microbial compositions between female and male nephrolithiasis patients. The metabolomic analysis identified 112 metabolites that were differentially expressed. Two significantly enriched metabolic pathways, including biosynthesis of unsaturated fatty acids and tryptophan metabolism, were also identified in nephrolithiasis patients. Four potentially diagnostic metabolites were also identified, including trans-3-hydroxycotinine, pyroglutamic acid, O-desmethylnaproxen, and FAHFA (16:0/18:2), and could function as biomarkers for the early diagnosis of nephrolithiasis. We also identified three metabolites that contributed to kidney stone size. Finally, our integrative analysis of the urinary tract microbiome and metabolome identified distinctly different network characteristics between the two groups.

**Conclusions:**

Our study has characterized important profiles and correlations among urinary tract microbiomes and metabolomes in nephrolithiasis patients for the first time. These results shed new light on the pathogenesis of nephrolithiasis and could provide early clinical biomarkers for diagnosing the disease.

## Introduction

Global incidences of nephrolithiasis are increasing annually (Zeng et al., 2017). Kidney stones are diagnosed by urinary ultrasound; however, patients are typically not diagnosed until the onset of urinary symptoms, such as renal colic, hematuria, and urinary infection. Without prompt treatment, nephrolithiasis may result in urinary tract obstruction and renal function insufficiency, resulting in considerable suffering and an economic burden on patients. Therefore, developing novel screening approaches and adopting early intervention will considerably improve outcomes for patients with nephrolithiasis.

In recent years, the microbiome has been shown to be involved in maintaining homeostasis and pathological processes of disease. Traditionally, the urinary system is considered sterile, and previous studies of nephrolithiasis have focused more on the gut microbiome (Liu et al., 2020b; Stanford et al., 2020). Observations from these previous studies have identified substantial differences in gut microbiota compositions between healthy individuals and nephrolithiasis patients (Stern et al., 2016). More recently, due to technological advances such as 16S RNA sequencing, studies have shown the presence of a urinary microbiome in healthy individuals (Wolfe and Brubaker, 2015; Karstens et al., 2018). Moreover, multiple research groups have demonstrated that urinary microbiomes were closely related to urological disorders, such as urinary tract infections and urinary incontinence (Nienhouse et al., 2014; Pearce et al., 2014). These studies indicate a role for the urinary microbiome in urological disorders. Several studies have also illustrated that Oxalobacter formigenes and Lactic acid bacteria alleviated nephrolithiasis by degrading oxalate (Sadaf et al., 2017; Chamberlain et al., 2020). A recent study has also determined that the urinary microbiome bears a stronger correlation with kidney stone disease than the gut microbiome (Zampini et al., 2019). Xie et al. illustrated a dysregulation of the urinary microbiota in men with calcium-based nephrolithiasis (Xie et al., 2020). These studies suggest that urinary microbiomes contribute to nephrolithiasis’s pathophysiology.

Kidney stone formation is strongly associated with metabolism, indicating an essential role of metabolites (Khan et al., 2016). Amino acids, urates, and purine have each been reported to influence the formation of kidney stones (Khan et al., 2016). Metabolites are not only the outputs of microbial metabolism but also function as biological effectors. Thus, metabolomics by next-generation sequencing offers a promising approach for determining disease biomarkers and developing therapeutic strategies. Duan et al. (YEAR) identified 15 metabolites and four metabolic pathways for nephrolithiasis using ^1^H nuclear magnetic resonance (NMR) spectroscopy (Duan et al., 2020). In another study, metabolic differences were analyzed in children with CaOx urolithiasis (Denburg et al., 2020). Although several of these studies have investigated the profiles of nephrolithiasis-associated metabolomes, studies characterizing the interplay between urinary microbiomes and metabolomes are still limited. Therefore, we aimed to elucidate the composition of microbial communities and metabolites of urinary microbiomes and metabolomes in healthy individuals and patients with kidney stones to provide novel targets for the early detection and intervention of nephrolithiasis.

Urine sampling is non-invasive, reliable, low-cost, and is commonly used in clinics for disease diagnosis and monitoring. To our knowledge, this is the first report to conduct an integrative analysis on the microbiome and metabolome in urine utilizing a multi-omic approach from healthy individuals and nephrolithiasis patients. The present study aimed to 1) characterize the urinary microbiome and metabolome in nephrolithiasis patients, 2) identify biomarkers for the early diagnosis of nephrolithiasis, and 3) investigate the potential correlation among the microbiota, metabolites, and clinical characteristics of nephrolithiasis.

## Materials and methods

### Study design and participants and sample collection

This study was approved by the Ethics Service Committee of the Zhujiang Hospital, Southern Medical University (China). The samples for this study were drawn from patients who had a physical check-up and diagnosed nephrolithiasis at the Zhujiang Hospital, Southern Medical University between July 2020 and January 2021. The physical check-up tern was defined as normal control (NC) group (n=9), the patients diagnosed nephrolithiasis were defined as nephrolith (NL) group (n=19). For NC group, individuals with personal history of urinary stones were excluded. For all participants, individuals with acute or chronic kidney disease, antibiotic use within 3 month and other chronic disease such as diabetes and hypertension were excluded. Their clinical feature including age, gender, body mass index (BMI), history of smoking and drinking, uric acid and urine PH were recorded. All participants have signed informed consent. The urine was collected form clean catch midstream, and 5 ml of urine was retained. All samples were placed on ice immediately before being snap-frozen and stored at -80°C within 5 minutes of collection.

### 16S rRNA gene sequencing and data analysis

One sample was eliminated from the NL group due to an inadequate sample volume. Overall, nine healthy participants remained in the NC group, and 18 nephrolith patients remained in the NL group. Each participant’s urine sample was analyzed by 16S rRNA gene sequencing. DNA extraction from samples was performed using the CTAB/SDS method. DNA concentration and purity were monitored on 1% agarose gels. Due to the inadequate urine volume, one sample was eliminated from NL group. Therefore, only 9 healthy participants (NC group) and 18 nephrolith patients (NL group) were devoted for 16S rRNA gene sequencing. The 16S rRNA gene V3–V4 variable region polymerase chain reaction (PCR) primers 515/806 with a barcode on the forward primer were used in a 30-cycle PCR using the Phusion^®^ High-Fidelity PCR Master Mix with GC Buffer (New England Biolabs). Primers targeting the V4 hypervariable region were used for PCR; FOR (5’-GTGCCAGCMGCCGCGGTAA-3’), REV (5’-GGACTACHVGGGTWTCTAAT-3’). PCR products were examined by 2% agarose gel electrophoresis in TBE and were recovered using the Qiagen gel extraction kit (Qiagen, Valencia, CA). Following purification and quantification, amplicons were pooled and sequenced by NovaSeq 6000 (Illumina). Raw paired-end reads were assembled and quality filtered to obtain clean data. All reads with more than 97% similarity were assigned to one operational taxonomic unit (OTU). Representative sequences of each OTU were aligned for classification. Species annotation of OTUs was applied using the Mothur method and SILVA138 database. Alpha diversity was estimated by Simpson’s index, Shannon’s index, and Chao1, and observed species indices were calculated to identify the diversity and evenness of species distributions of samples using Qiime software (Version 1.9.1). Rarefaction curves were generated to evaluate the richness of OTUs, and rank abundance curves were generated to assess the evenness of all samples using R software (Version 4.0). Welch’s t-test compared Between-group differences in β diversity, including unweighted and weighted UniFrac distances. Phylogenetic distribution of microbiota between NC and NL groups was presented by circle plot. Potential microbial biomarkers closely associated with the two groups were discriminated by LEfSe software with a linear discriminant analysis (LDA) score threshold of 2.5.

### Untargeted metabolomics and analysis

Urine from the nine healthy participants (NC group) and 19 nephrolith patients (NL group) were analyzed by metabolomics using liquid chromatography-mass spectroscopy (LC-MS). Urine samples were collected in Eppendorf tubes and homogenized with 80% methanol. The mixtures were incubated for 5 min at 4°C and centrifuged for 20 min at 15000 g and 4°C. Supernatants were collected for dilution in LC-MS grade water. The supernatants were collected and used for the LC-MS analysis following centrifuging. Samples of equal volume from each sample was mixed as a quality control sample, which was analyzed throughout the experiment to monitor the reliability and stability of the system. LC-MS analyses were performed by a Vanquish UHPLC system (ThermoFisher, Germany) coupled with an Orbitrap Q ExactiveTM HF mass spectrometer (Thermo Fisher, Germany) in Novogene Co., Ltd. (Beijing, China). Eluent A (0.1% FA in Water) and eluent B (Methanol) were employed as the eluents for the positive polarity mode, while eluent A (5 mM ammonium acetate, pH 9.0) and eluent B (Methanol) were applied as the eluents for the negative polarity mode. The gradient program was set as follows: 2% B, 1.5 min; 2-85% B, 3 min; 85-100% B, 10 min; 100-2% B, 10.1 min; 2% B, 12 min. Q ExactiveTM HF mass spectrometer was worked in positive or negative polarity mode. Raw data were analyzed using Compound Discoverer 3.1 software. Each metabolite was screened by peak alignment and peak picking. After qualitative, quantitative, and quality control analyses, a multivariate statistical analysis of the metabolites was performed. Partial least squares discriminant analysis (PLS-DA) was employed to reveal the differences in metabolic patterns. Metabolites were annotated using the Kyoto Encyclopedia of Genes and Genomes (KEGG) database. Metabolites with fold-changes (FC) ≥ 1.2 or ≤ 0.67 and variable importance in projection (VIP) scores > 1 (*P* < 0.05) were identified as differentially expressed metabolites (DEMs). DEMs were visualized with a volcano plot and heatmaps using the ggplot2. To evaluate the metabolite’s discrimination abilities, receiver operating characteristic curve (ROC) analysis was carried out using the pROC package in R. And area under the curve (AUC) was calculated. Correlation analyses between metabolites and clinical features were performed by Spearman’s correlation. Networks revealing correlations between microbes and metabolites were visualized with Cytoscape software (version 3.7.2).

### Statistical analysis

Clinical features were compared by Fisher’s exact test for categorical variables and Student’s t-test for the continuous variables. Differences in metabolic profiles were analyzed by PLS-DA, VIP, and Welch’s t-test. The correlation between metabolites and clinical features was investigated by Spearman’s correlation analysis. P-values less than 0.05 were considered significantly different.

## Results

### Characteristics of participants

A total of 19 nephrolithiasis patients and nine healthy controls were enrolled in the study. Of them, 27 samples were collected for microbiome analyses. The clinical characteristics of the 28 participants are shown in **Table 1**. Age, gender, BMI, urine pH, and lifestyle habits, including smoking and alcohol consumption, displayed no significant differences between the NC and NL groups. Though uric acid presented higher in nephrolithiasis patients, there was no significant difference (*P* = 0.133). No statistically significant differences were identified in the demographic and clinical variables between the two groups, indicating no major confounding factors exist within the experiment design.

**Table 1:** Clinical information of the participants.

Characteristics	NC group (n=9)	NL group (n=19)	*P*
Age, years	44.0 ± 14.8	52.3 ± 13.9	0.160
Gender (% female)	44.4%	31.6%	0.507
BMI, kg/m^2^	22.2 ± 3.2	23.5 ± 3.7	0.369
Smoke (n,%)	55.6%	47.4%	0.686
Drink (n,%)	55.6%	57.9%	0.907
Uric acid	339.3 ± 65.9	387.95 ± 82.0	0.133
Urine PH	6.9 ± 0.5	6.9 ± 0.4	0.976
Stone size (mm)	NA	14.1 ± 6.3	NA

NA, not applicable.

### Urinary microbial diversity and structure analysis

**Figure 1:**
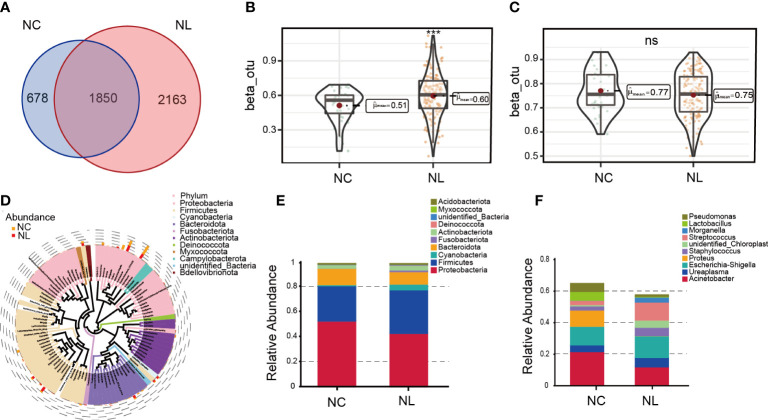
Urinary microbiome diversity and structure analysis. **(A)** Operational taxonomic units (OTUs) between the normal control (NC) and nephrolith (NL) groups. **(B, C)** Comparison of β diversity based on weighted and unweighted UniFrac distance between the NC and NL groups. **(D)** Cladogram presenting phylogenetic distribution of microbiota between the NC and NL groups. **(E)** Relative abundances of bacterial phyla between NC and NL groups. **(F)** Relative abundances of bacterial genera between NC and NL groups. ***P < 0.001. ns, no significance.

A total of 2,342,215 high-quality sequencing reads were obtained after splicing and filtering. Following taxonomic assignment, 4,720 OTUs were clustered. Rarefaction curves indicated sufficient sequencing data was achieved for the study (**Figure S1A**), while the rank abundance curves reflected an evenness across all samples (**Figure S1B**). Additionally, the accumulation maps of abundance suggested that the sample size in our study for 16S rRNA gene sequencing was adequate (**Figure S1C**). To determine the characterization of the urinary microbiome in two groups, α and β diversity were calculated. No significant differences were found in α diversity determined by Simpson, Shannon, Chao1 or observed species indices (**Figure S2A–D**). The Venn diagram (**Figure 1A**) shows 2,163 unique features in the NC group, while 678 unique features were found in the NL group. The overlaps identified 1,850 common features. β diversity was assessed to determine the similarity of the microbial community structures between the two groups. As a result, the two groups presented a significant difference in microbial communities based on weighted UniFrac analysis, while no significant difference was observed by unweighted UniFrac analysis (**Figures 1B, C**). Sequences from all samples were categorized into seven taxonomic classification levels, including two kingdoms, 59 phyla, 134 classes, 285 orders, 423 families, 718 genera, and 383 species (**Table S1**). A phylogenetic tree was constructed using the OTUs. The circle plot (**Figure 1D**) depicts the classification level from phylum to genus visualizing from inside to outside. The relative abundances of dominant taxa were assessed by microbial taxon assignment (**Figures 1E, F**, **Figure S3**). Proteobacteria was the most abundant phylum, representing 52.4% and 42.6% of the OTUs in the NC and NL groups, respectively (**Figure S3A**). The second most abundant phylum in both the NC and NL groups was Firmicutes (28.2% versus 34.6%, respectively) (**Figure S3A**). At the genus level, Streptococcus was enriched in the NL group, whereas Lactobacillus was enriched in the NC group (**Figure S3E**). Escherichia coli accounted for a greater proportion in the NL group than the NC group (32.64% versus 24.03% respectively) at the species level (**Figure S3F**). These results revealed that microbial diversity and composition in nephrolithiasis patients vary significantly.

### Alteration of the urinary microbiome in nephrolithiasis

**Figure 2:**
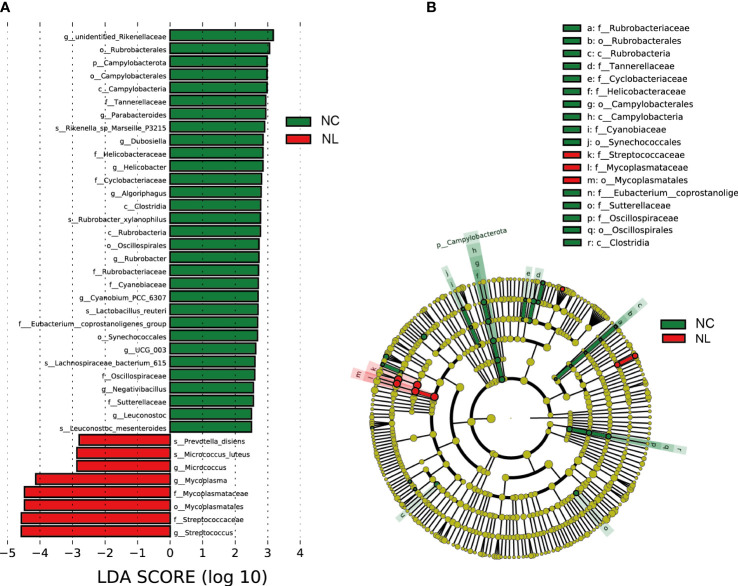
Urinary microbiome differential analysis. **(A)** Difference in abundance estimated by linear discriminant analysis effect size (LEfSe) between the normal control (NC) and nephrolith (NL) groups. **(B)** Taxonomic cladogram presenting phylogenetic distribution of microbiota related to NC and NL groups. Threshold of linear discriminant analysis (LDA) greater than 2.5 are shown.

LEfSe was applied to identify differentially abundant taxa with LDA scores greater than 2.5 in two groups. In total, 39 discriminative taxa were identified (**Figure 2A**). Higher levels of Streptococcus, Streptococcaceae, and Mycoplasmatales were found in the NL group, while the NC group mainly displayed higher enrichment in Rubrobacterales, Campylobacterota, Parabacteroides, and Lactobaccilus. In the NL group, 31 taxa were enriched, whereas eight taxa were enriched in the NC group. The cladogram (**Figure 2B**) distinctly depicted the hierarchical correlation of these discriminative microbes in both the NC and NL groups. These results suggest that a variation in urinary microbiomes could be an important factor in nephrolithiasis formation.

### Comparison of urinary microbiome between female and male

We performed a comparative analysis of the urinary microbiomes between females and males to determine whether sex specific variations in their compositions were present. The gender comparison (**Figure S4A**) showed that Simpson’s index α diversity in males was lower than in females (*P*<0.05). β diversity based on the binary Jaccard distance and Bray Curtis distance revealed a significant difference between females and males (**Figures S4B, C**), indicating a significant difference in microbiota diversity between sexes. The LEfSe analysis (LDA) showed that Alphaproteobacteria, Actinomycetaceae, and Erysipelotrichaceae were more abundant in males, while the Prevotellaceae, Morganellaceae, Fusobacteriaceae, Peptostreptococcaceae, Aerococcaceae and Veillonellaceae families were all overrepresented in females (**Figure S4D**).

### Comparison of midstream urinary culture and 16S rRNA gene sequencing

Results of midstream urinary culture revealed that 11 samples had positive urine cultures out of 18 samples, while the remaining seven samples all generated positive results by 16S rRNA gene sequencing. Almost all urinary culture results could be found in 16S rRNA gene sequencing except for two samples presenting mixed bacterial flora. Escherichia-Shigella and Prevotella were the dominant groups of the two mixed samples, accounting for 95.5% and 35.7% of the sequence reads, respectively. The bacteria identified by urinary culture were not necessarily the dominant bacteria identified by 16S rRNA sequencing. In two samples, E. coli were positively identified by urinary culture, but the corresponding proportions detected by sequencing were 2.0% and 0.8%, respectively. Our results showed that the positive rate of urinary 16s rRNA sequencing was 100%, indicating a robust discriminative capacity.

### Alteration of the urinary metabolome in nephrolithiasis

**Figure 3:**
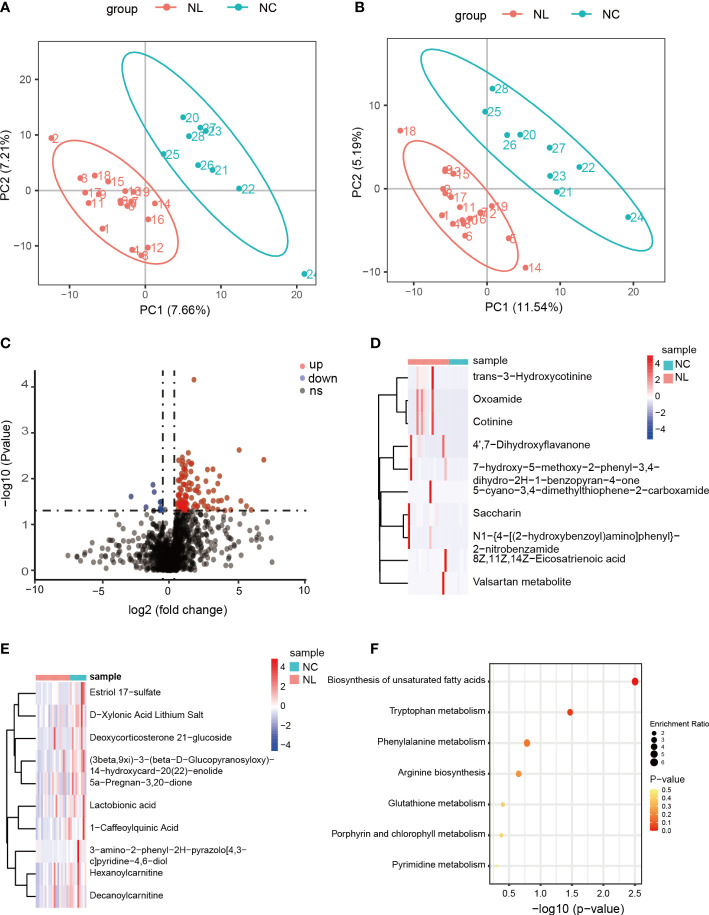
Differentially expressed metabolites between control individuals and nephrolithiasis patients. **(A)** Partial least squares discriminant analysis (PLS-DA) of positively charged metabolites. **(B)** PLS-DA of negatively charged metabolites. **(C)** Volcano plot of differentially expressed metabolites (DEMs), characterized by fold change ≥ 1.2 or ≤ 0.667 (P < 0.05) with a threshold of VIP ≥ 1. Red dots represent up-regulated metabolites, blue dots represent down-regulated metabolites. **(D)** Heatmap of top 10 up-regulated metabolites in nephrolith patients. **(E)** Heatmap of top ten down-regulated metabolites in nephrolith patients. **(F)** KEGG enrichment analysis of the DEMs.

We carried out a metabolome analysis to determine metabolite variations among the groups using an LC-MS-based metabolomics approach. In total, 1,477 metabolites were identified and were mapped using the KEGG metabolic pathways, such as lipid metabolism, amino acid metabolism, and carbohydrate metabolism (**Figure S5**). PLS-DA was performed to evaluate the ability of positively and negatively charged metabolites for classifying. The PLS-DA analysis distinguished two clear groups among the samples (**Figures 3A, B**), indicating variations in the urinary metabolomes between the NC and NL groups. The 112 identified DEMs were visualized by a volcano plot (**Figure 3C**), of which 102 DEMs were up-regulated, and ten were down-regulated. The top ten up-regulated DEMs in the NC and NL groups are displayed in **Figures 3D, E**, respectively. The abundant metabolites in the NC group were mainly lactobionic acid, 1-caffeoylquinic acid, and deoxycorticosterone 21-glucoside. Moreover, the abundance of stearic acid, arachidic acid, homocysteic acid, gluconic acid, and serotonin were significantly increased in the NL group only, indicating distinct metabolite compositions between the NC and NL groups. Furthermore, the DEMs KEGG pathway analysis identified significant enrichment in biosynthesis of unsaturated fatty acids and tryptophan metabolism pathways.

### Identification of biomarkers for nephrolithiasis

**Figure 4:**
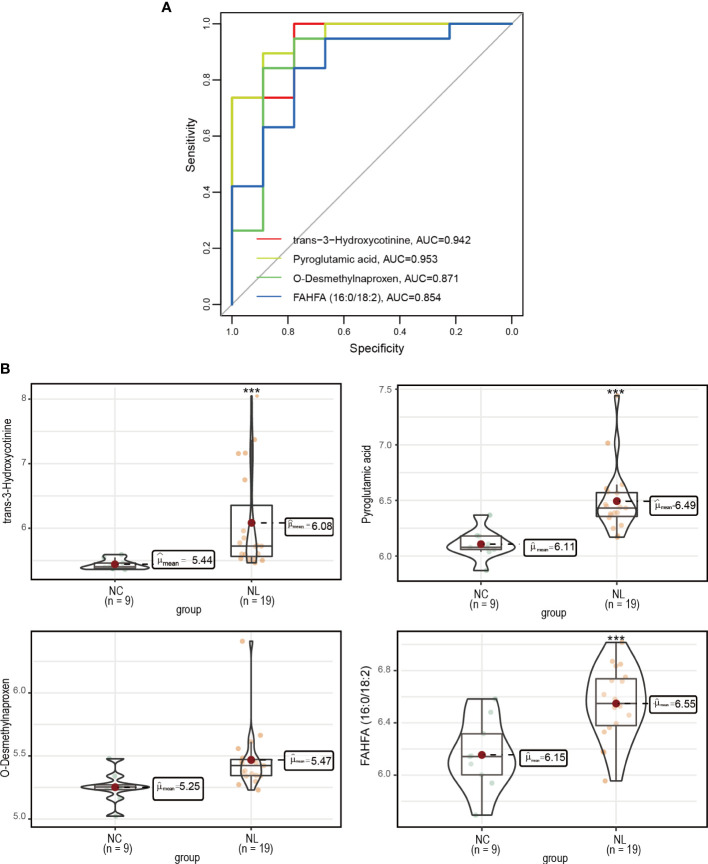
Measurement of potential biomarkers. **(A)** Performance assessment of nephrolithiasis prediction base on trans-3-hydroxycotinine, pyroglutamic acid, O-desmethylnaproxen and FAHFA (16:0/18:2) levels. **(B)** The concentrations of trans-3-hydroxycotinine, pyroglutamic acid, O-desmethylnaproxen and FAHFA (16:0/18:2) between control individuals and nephrolithiasis patients. Normal control (NC), nephrolith (NL). ***P < 0.001.

We employed a ROC analysis of the DEMs to evaluate their predictive ability of nephrolithiasis. As a result, four DEMs, trans-3-hydroxycotinine, pyroglutamic acid, O-desmethylnaproxen, and FAHFA (16:0/18:2) presented AUC values greater than 0.85 (**Figure 4A**), indicating good diagnostic abilities. We further investigated the abundance of these four metabolites in the NC and NL groups (**Figure 4B**), confirming all four metabolites exhibited a higher abundance in the NL group than the NC group.

### Correlation analysis between metabolites and clinical features

**Figure 5:**
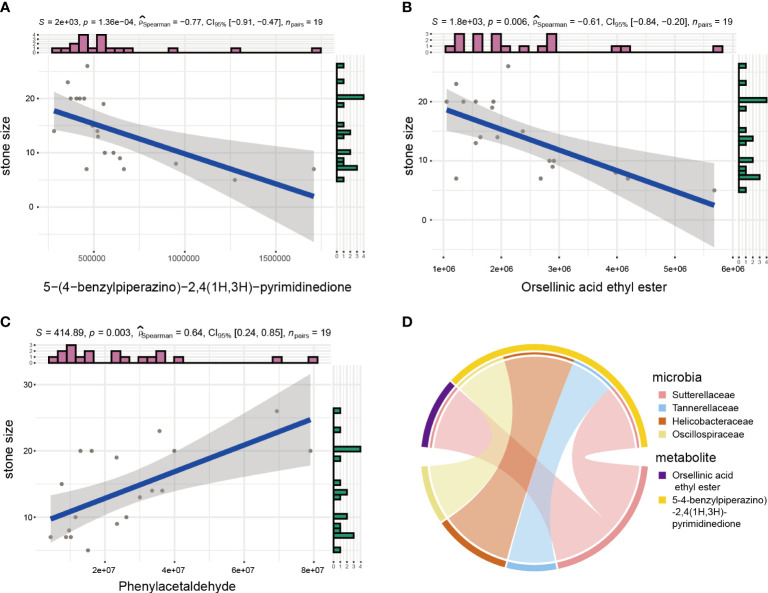
Microbial and metabolite correction analyses. **(A–C)** Spearman analysis between stone size and urinary metabolites. **(D)** Circos plot showing correlation between microbes and metabolites related to stone size.

Kidney stone detection and nephrolithiasis treatment are both affected by stone size. Identifying correlations between metabolites and stone size may help clinical diagnosis and treatment. Thus, correlations between metabolites and stone size were calculated by Spearman’s correlation coefficient, which identified three metabolites with correlation coefficients greater than 0.6. Among them, 5-(4-benzylpiperazino)-2,4(1H,3H)-pyrimidinedione and Orsellinic acid ethyl ester were significantly negatively correlated with stone size, while Phenylacetaldehyde was positively correlated with it (**Figure 5A–C**). Subsequently, we investigated whether there are any microbes were correlated with these three metabolites. So we performed correlation analyses between microbes and these three metabolites (**Figure 5D**). And the results showed that the change in abundance of Sutterellaceae was positively correlated with orsellinic acid ethyl ester. In addition, the changes in abundance of Tannerellaceae, Helicobacteraceae and Oscillospiraceae were also positively correlated with 5-(4-benzylpiperazino)-2,4(1H,3H)-pyrimidinedione.

### Correlation analysis between microbes and metabolites

**Figure 6:**
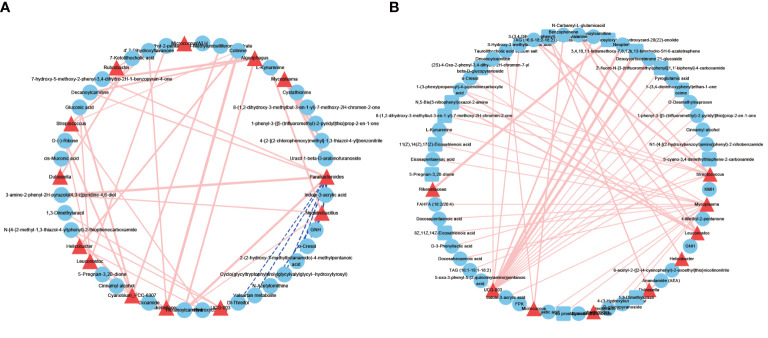
Network profiles between microbes and metabolites. **(A)** A network profile of the normal control group. **(B)** A network profile of the nephrolith group. Red line indicate positive correlations, dashed blue lines represent negative correlations.

As microbes can produce various bioactive metabolites, we investigated the possible correlation between urinary microbes and metabolites (**Figure 6**). The correlations between microbes and metabolites in the NL group were all positively correlated. We found that the correlation between microbes and metabolites in the NL group was stronger than that in the NC group, indicating that these interactions might be responsible for the highly abundant metabolites in the disease group. Several genera had greater interactions or stronger relationships in the NL group than the NC group. For example, Clostridia UCG-003 and Streptococcus were more closely correlated with metabolites in the NL group, indicating an important role in metabolite generation associated with kidney stone formation. Notably, Clostridia UCG-003 was positively correlated with O-desmethylnaproxen (r = 0.99, *P* < 0.05), pyroglutamic acid (r = 0.95, *P* < 0.05), and neopterin (r = 0.9, *P* < 0.05) in the NL group. Moreover, we have identified O-desmethylnaproxen and pyroglutamic acid as biomarkers for nephrolithiasis, while Clostridia UCG-003 specifically might also be implicated in the formation of kidney stones.

## Discussion

Mounting evidence indicates an association between the microbiome and metabolome in kidney stone formation. Identifying urinary microbes and metabolites as potential biomarkers for early screening and intervention holds significant promise for nephrolithiasis prognosis and diagnosis. A past study of urinary stone disease found no significant difference in the microbial composition of urine between the lower and upper urinary tracts (Dornbier et al., 2020). Therefore, we elected to sample lower urinary tract urine as it is more convenient for collection. Previous studies have demonstrated that several clinical factors, such as BMI and alcohol consumption, were associated with nephrolithiasis (Khalili et al., 2021; Lee et al., 2022). In our study, clinical characteristics, including age, gender, BMI, urine pH, smoking habits, and alcohol consumption, between the NC and NL groups showed no statistically significant differences, excluding potential confounding factors. Notably, microbes were found abundantly in urine samples using 16S rRNA gene sequencing, which performed better than traditional midstream urinary culture. The 16S rRNA gene sequencing attained a higher positive rate than urinary culture, while the bacteria identified by urinary culture were not the dominant bacteria identified by 16S rRNA gene sequencing. The differences in bacterial identification between the two methods may be due to the growth limitations of routine culturing methods. Therefore, considerably more information about bacterial community compositions was available through 16S rRNA gene sequencing, which may help formulate better treatment protocols.

Microbial richness was closely associated with nephrolithiasis. Previous studies have demonstrated that E. coli contributes to kidney stone formation (Tavichakorntrakool et al., 2012; Tavichakorntrakool et al., 2017). In one gut microbiome study, E. coli was identified as being a powerful predictor for nephrolithiasis (Tang et al., 2018). Consistent with their reports, we found that E. coli also accounted for a greater proportion of the microbiomes in nephrolithiasis patients than in healthy individuals. Additionally, previous studies have determined that the genus Staphylococcus was overrepresented, while Lactobacillus was less abundant in nephrolithiasis patients than in healthy controls (Zampini et al., 2019; Liu et al., 2020a; Kachroo et al., 2021); again, our findings support these reports. It has been shown that Staphylococcus promotes the formation of kidney stones by producing the enzyme urease, which increases pH, while Lactobacilli exerts an inhibitory action by degrading oxalate (Lewanika et al., 2007; Torzewska et al., 2019). We report for the first time the involvement of mycoplasma and micrococcus in nephrolithiasis, this is a significant finding, and their potential implications in stone formation should be studied in greater detail in future studies.

Previous studies have also reported that males develop nephrolithiasis with higher incidences than females (Zeng et al., 2017; Zhu et al., 2019). To determine whether this difference was associated with their microbiomes, we performed a compositional analysis of the urinary microbiota between males and females with nephrolithiasis. Notably, both α and β diversity presented a statistically significant difference between males and females, suggesting the mocrobiota might contribute to the difference in case incidences. Lachnospiraceae, Prevotellaceae, and Peptostreptococcaceae were overrepresented in females and are considered potentially beneficial bacterial families. We speculate that the prevalence of nephrolithiasis differed between the sexes because of the increase of beneficial bacterial families in females, which required more studies for validation.

Importantly, imbalances in urinary microbiota may initiate the dysregulation of metabolites. In parallel with the results of the urinary microbiome, urinary metabolite composition changed considerably. The PLS-DA analysis identified a distinct variation in metabolites between the NC and NL groups; in total, 112 metabolites were identified as DEMs. We identified four metabolites with promising diagnostic potential: pyroglutamic acid, trans-3-hydroxycotinine, O-desmethylnaproxen and FAHFA (16:0/18:2). It has been reported that the accumulation of pyroglutamic acid could result in metabolic acidosis, which might promote stone formation (Wagner and Mohebbi, 2010; Weiler et al., 2015). The other three metabolites were found to correlated with nicotine metabolism, anti-inflammation, and lipid metabolism (Tan et al., 2019; Dator et al., 2020; Wojcieszyńska and Guzik, 2020). However, no related studies have reported the relationship between these metabolites and kidney stones, which should be investigated further to characterize their roles in the disease.

Significant enrichment in biosynthesis of unsaturated fatty acids and tryptophan metabolism pathways were identified by KEGG analysis. Arachidic acid, an omega-6 fatty acid, is an inflammatory mediator, which may be associated with the formation of kidney stones (Rodgers and Siener, 2020). However, the role of other unsaturated fatty acids, including docosahexaenoic acid and eicosapentaenoic acid, remains unclear (Taylor et al., 2005; Siener et al., 2011; Rodgers et al., 2019). Approximately 95% of Tryptophan is metabolized by the kynurenine pathway, which may contribute to pro-oxidative action and the inflammatory response in kidney disease (Wang et al., 2015; Hsu and Tain, 2020). Previous studies found that oxidative stress and inflammation may correlate with nephrolithiasis (Dominguez-Gutierrez et al., 2018; Sharma et al., 2019), indicating a possible role for Tryptophan metabolism in kidney stone formation.

We explored the effect of metabolites on stone size. The results demonstrated that 5-(4-benzylpiperazino)-2, 4(1H,3H)-pyrimidinedion, and orsellinic acid ethyl ester were negatively correlated with stone size, whereas Phenylacetaldehyde was positively correlated, indicating that these metabolites inhibited or promoted stone growth. In general, the results from our nontargeted metabolomics analysis identified unique metabolic signatures, enabling a better understanding of the pathogenesis of nephrolithiasis.

Microbes may exert effects on catabolite or anabolism of metabolites, and correspondingly, metabolites could facilitate or inhibit microbe growth dynamics. We constructed microbe-metabolite networks by integrating urinary microbiomes and metabolomes, and the networks generated identified a statistically significant difference between the two groups. A stronger association between microbes and metabolites was found in the NL group than the NC group, indicating that the highly enriched metabolites in the NL group may contribute to the imbalanced microbiotas or their interactions. Clostridia UCG-003 and Streptococcus had more interactions and a stronger association with metabolites, suggesting a more central metabolic role in the NL group than the NC group. Of note, Streptococcus was also significantly enriched in the NL group. A multi-omics integration revealed more potential mechanisms regarding how *Streptococcus* contributes to stone formation. Investigating the relationships between microbes and metabolites has indicated the significance of their interactions and has provided an insightful perspective for further study.

As a preliminary analysis, several limitations in our study must be highlighted. First, our sample sizes were relatively small, which may affect the generalizability of our results. Second, we reported the characteristics and associations of microbes and metabolites in nephrolithiasis without attributing causality, which will require further investigation and validation.

Our study has determined distinct profiles of the urinary microbiome and metabolome in nephrolithiasis patients compared with healthy controls. In addition, we have identified novel biomarkers and enriched pathways for diagnosing stone formation or that may contribute to disease progression, respectively. Our study provides new insights into nephrolithiasis pathogenesis and provides potential targets for early intervention of the disease.

## Data availability statement

The 16s sequencing reads files were deposited into the Sequence Read Archive (SRA) of the National Center for Biotechnology Information (NCBI) with accession number SRP394450 under project PRJNA874352. https://www.ncbi.nlm.nih.gov/bioproject/PRJNA874352.

## Ethics statement

The studies involving human participants were reviewed and approved by University of Chinese Academy of Sciences Shenzhen Hospital (LL-TK-2020109). The patients/participants provided their written informed consent to participate in this study.

## Author contributions

Data analysis were performed by HG and JL. The manuscript was written and drafted by FX and YH. Sample collection was performed by ZY. Data designed were performed by SP. All authors read and approved the final manuscript.

## Funding

This study was funded by Health system scientific research project of Guangming district, Shenzhen City (2020R01040) and the Nation Key R&D Program of China (2019YFC0121904).

## Conflict of interest

The authors declare that the research was conducted in the absence of any commercial or financial relationships that could be construed as a potential conflict of interest.

## Publisher’s note

All claims expressed in this article are solely those of the authors and do not necessarily represent those of their affiliated organizations, or those of the publisher, the editors and the reviewers. Any product that may be evaluated in this article, or claim that may be made by its manufacturer, is not guaranteed or endorsed by the publisher.
