# Supplementary material for: Urinary Microbial and Metabolomic Profiles in Kidney Stone Disease
Source: Front Cell Infect Microbiol. 2022 Sep 5;12:953392. doi: 10.3389/fcimb.2022.953392 (PMC9484321; doi:10.3389/fcimb.2022.953392)
Supplement: Supplementary file 1 [file DataSheet_1.pdf]

Supporting information

Table S1 Taxonomic composition of urinary microbiome of samples

microbial taxonomic	kingdom	phylum	class	order	family	genus	species
number	2	59	134	285	423	718	383

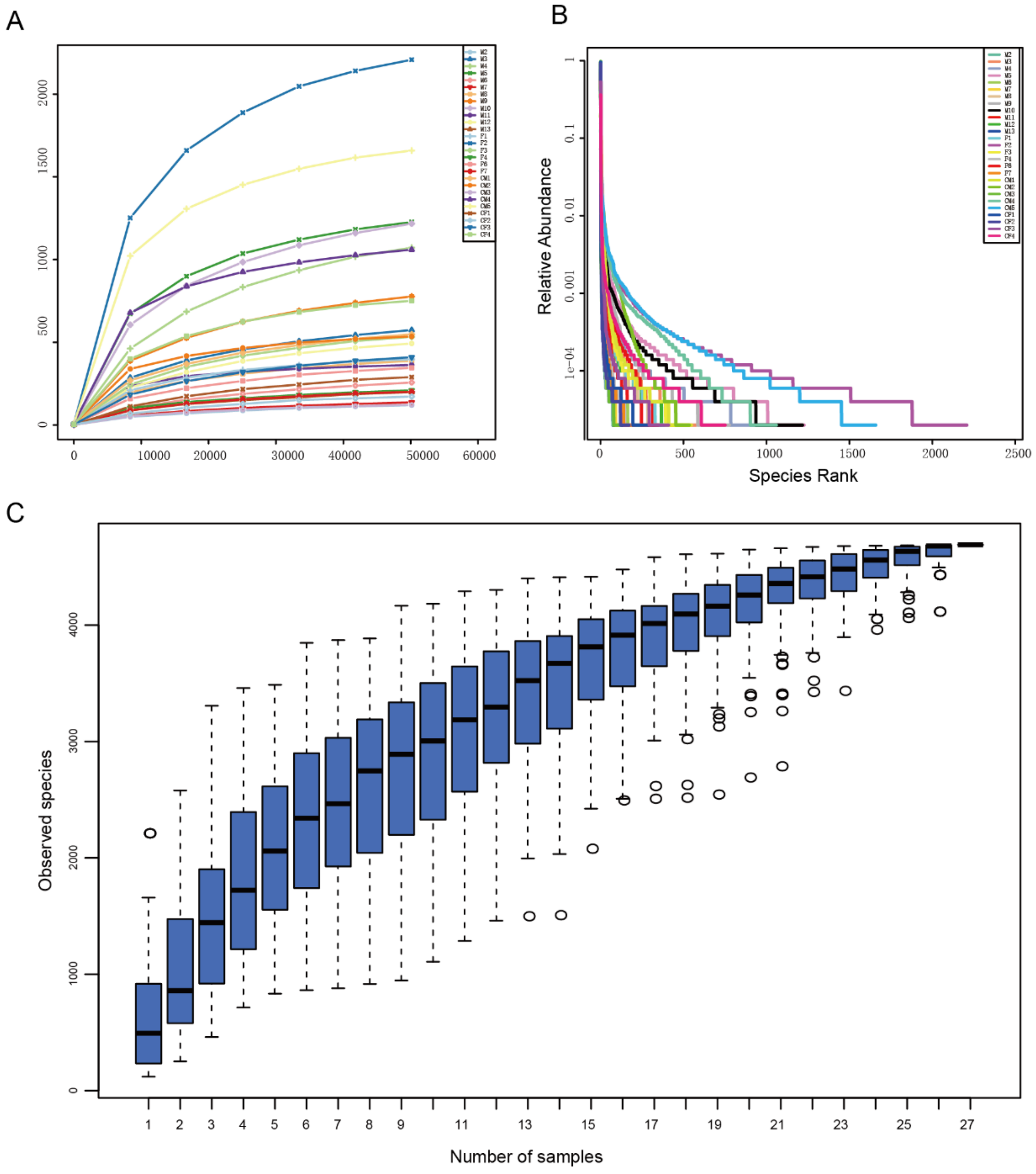

Fig S1 Abundance curves estimating sample evenness and richness. (A) Rarefaction curves. (B) Rank abundance curves. (C) Abundance accumulation maps.

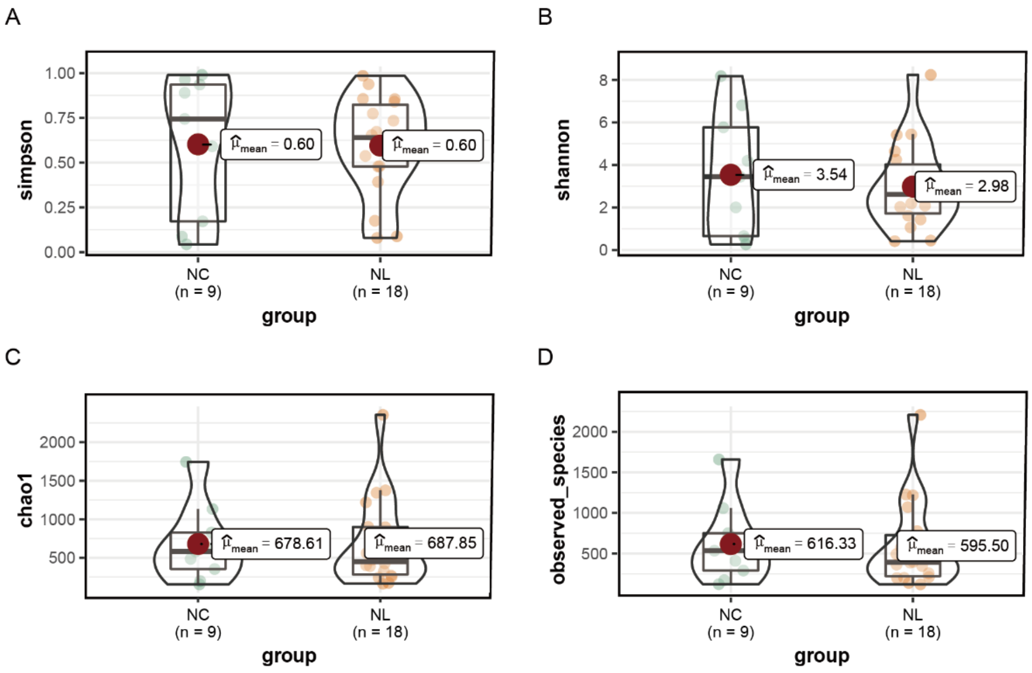

Fig S2 Comparison of α diversity was evaluated by simpson (A), shannon (B), chao1 (C) and observed species (D).

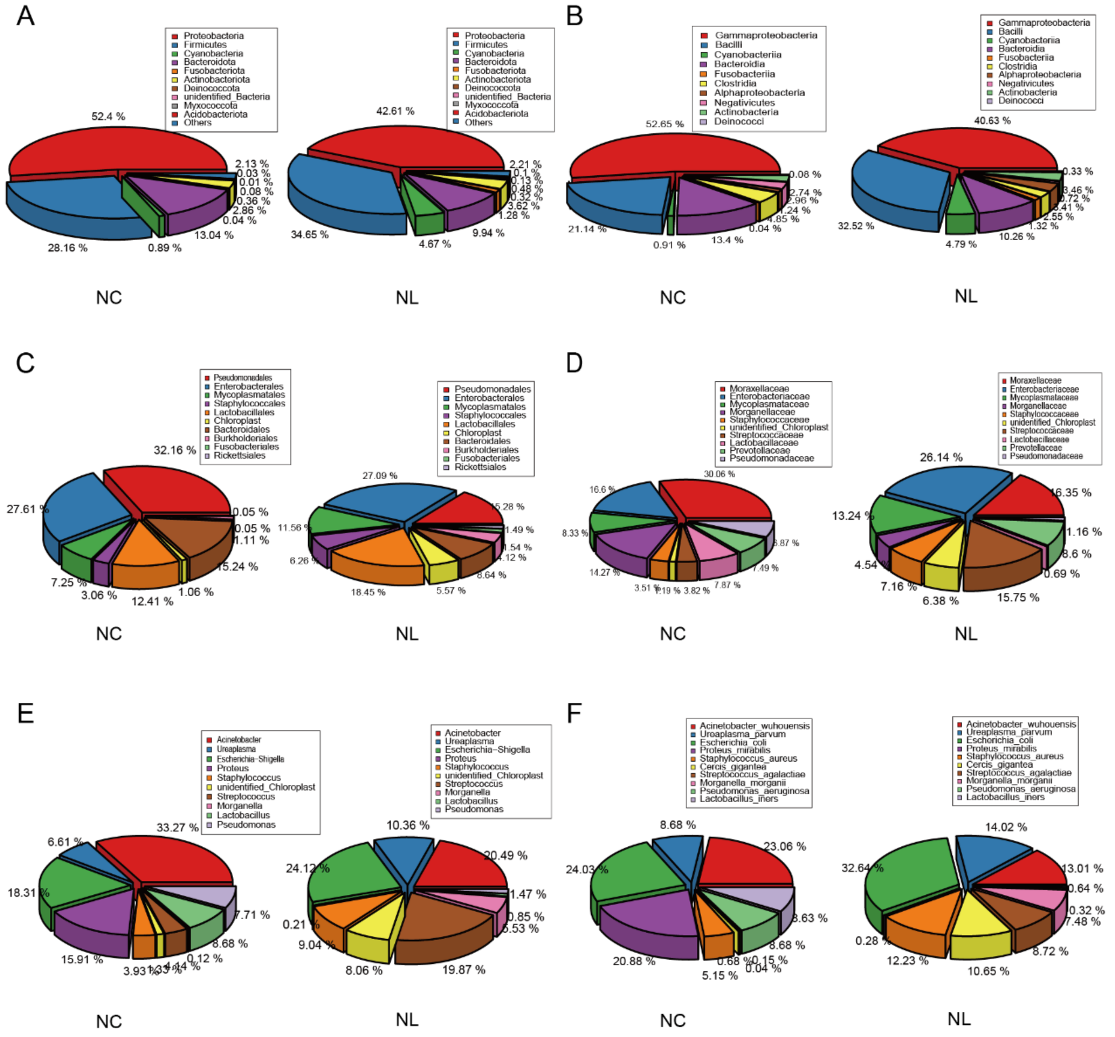

Fig S3 Pie charts of relative abundance of bacteria. Pie chart showing relative proportion of bacterial phylum (A), class (B), order (C), family (D), genus (E) and species (F) in NC and NL groups.

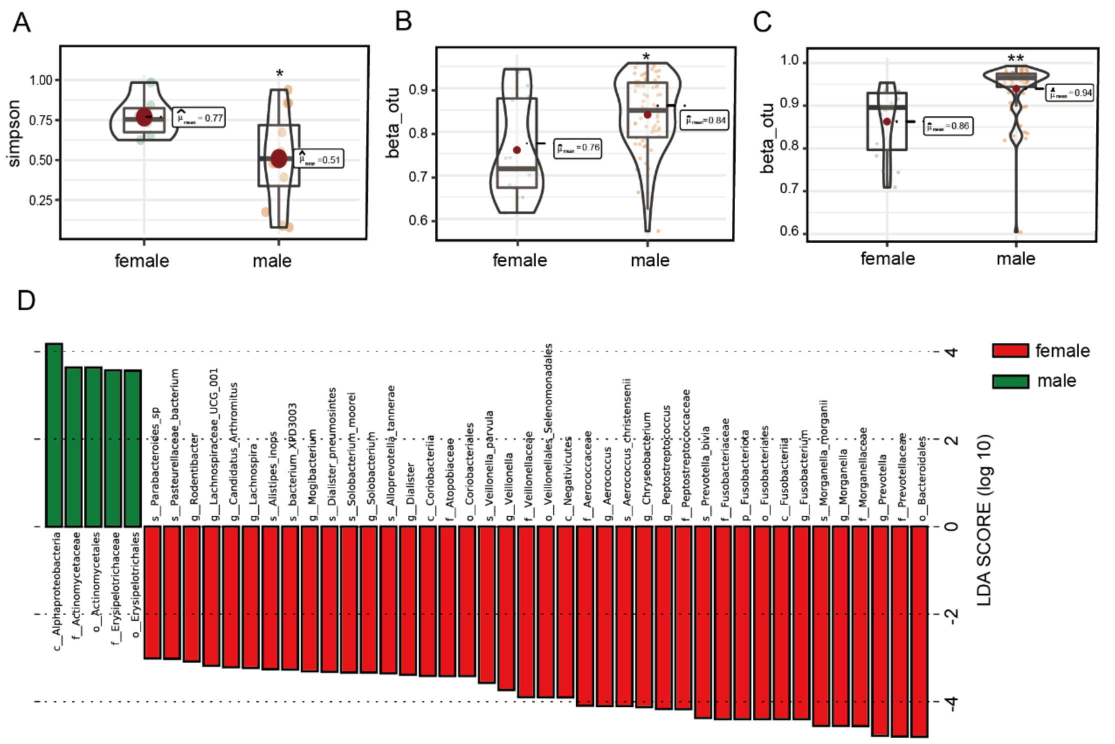

Fig S4 Analysis of urinary microbiome between female and male. (A) α diversity evaluated by simpson. (B) Comparison of β diversity based on binary jaccrd distance. (C) Comparison of β diversity based on bray curtis distance. (D) Estimation of difference in abundance by linear discriminant analysis effect size (LEfSe). Threshold of linear discriminant analysis (LDA) more than 3 were showed. **P*<0.05. ***P*<0.01.

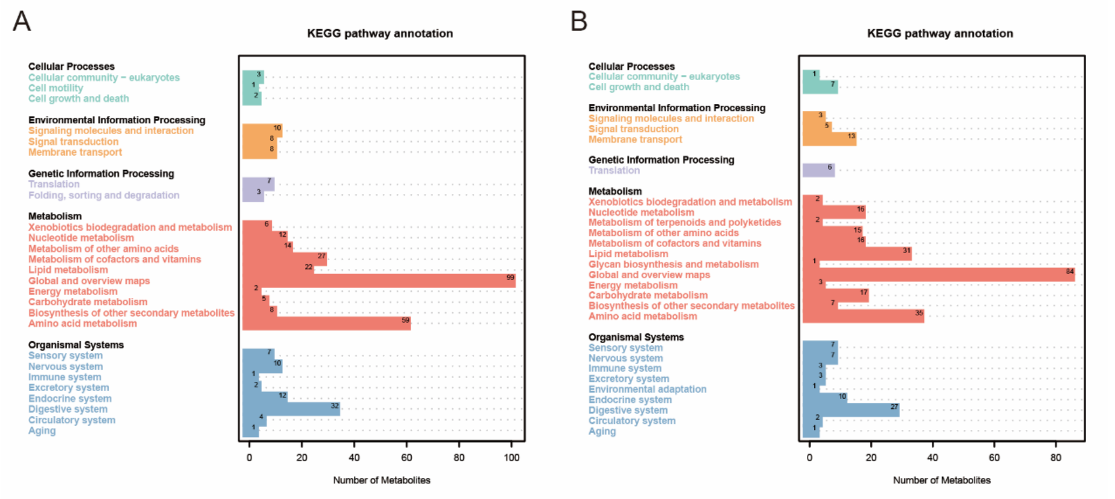

Fig S5 Metabolites annotation by KEGG. (A) Annotation of positively charged metabolites. (B) Annotation of negatively charged metabolites.
